# Dynamic placement of the linker histone H1 associated with nucleosome arrangement and gene transcription in early *Drosophila* embryonic development

**DOI:** 10.1038/s41419-018-0819-z

**Published:** 2018-07-09

**Authors:** Jian Hu, Liang Gu, Youqiong Ye, Meizhu Zheng, Zhu Xu, Jing Lin, Yanhua Du, Mengxue Tian, Lifang Luo, Beibei Wang, Xiaobai Zhang, Zhiping Weng, Cizhong Jiang

**Affiliations:** 1 Institute of Translational Research, Tongji Hospital, the School of Life Sciences and Technology, Shanghai Key Laboratory of Signaling and Disease Research, the Collaborative Innovation Center for Brain Science, Tongji University, Shanghai, 200092 China; 2 Department of laboratory medicine, the first people’s Hospital of Ninghai County, Ningbo city, 315600 China; 3 Program in Bioinformatics and Integrative Biology, University of Massachusetts Medical School, Worcester, MA 01605 USA

## Abstract

The linker histone H1 is critical to maintenance of higher-order chromatin structures and to gene expression regulation. However, H1 dynamics and its functions in embryonic development remain unresolved. Here, we profiled gene expression, nucleosome positions, and H1 locations in early *Drosophila* embryos. The results show that H1 binding is positively correlated with the stability of beads-on-a-string nucleosome organization likely through stabilizing nucleosome positioning and maintaining nucleosome spacing. Strikingly, nucleosomes with H1 placement deviating to the left or the right relative to the dyad shift to the left or the right, respectively, during early *Drosophila* embryonic development. H1 occupancy on genic nucleosomes is inversely correlated with nucleosome distance to the transcription start sites. This inverse correlation reduces as gene transcription levels decrease. Additionally, H1 occupancy is lower at the 5′ border of genic nucleosomes than that at the 3′ border. This asymmetrical pattern of H1 occupancy on genic nucleosomes diminishes as gene transcription levels decrease. These findings shed new lights into how H1 placement dynamics correlates with nucleosome positioning and gene transcription during early *Drosophila* embryonic development.

## Introduction

Eukaryotic genomic DNA is packaged into chromatin through the formation of nucleosome. The nucleosome is the basic repeating unit of eukaryotic chromatin, consisting of an octamer of histones with two copies of each of histone proteins H2A, H2B, H3, and H4, around which ~146 bp of DNA is wrapped in a left-handed toroid^1^. The linker histone H1 binds to nucleosome to form the chromatosome to further condense the chromatin structure^2^. Previous studies showed that the abundance of linker histones in the nucleus was about equal to that of the nucleosome in higher eukaryotes^3^. This suggests that H1 plays an important structural and functional role in chromatin. For example, the histone H1 is essential for the chromosome architecture and segregation in mitosis^4^. The binding of histone H1 to nucleosomes facilitates nucleosomal stabilization through the formation of linker histone/DNA stem structure^5,6^. Linker histones now appear to also exert functions in regulating fundamental biological processes, including gene expression^7^, stem cell differentiation^8^, and mouse embryonic development^9^. Thus, the precise in vivo roles of linker histones remain elusive.

Linker H1 histones typically comprises three parts: a central globular domain, a short N-terminal region, and a long unstructured C-terminal tail^10^. The N-terminal region is not important for H1 binding to nucleosome^6^, whereas the C-terminal tail is required for H1 binding to chromatin in vivo^11^. However, the globular domain alone is sufficient for chromatosome formation and protects the same linker DNA in the native chromatin against micrococcal nuclease digestion as the full-length linker histone^10,12^. The studies of nucleosome recognition by linker histones have led to many conflicting models for how the globular domain binds to the nucleosome. Previous studies suggested that linker histones bound to the nucleosome on^6,12,13^ or off^14,15^ the dyad. Whether the globular domain interacts with both or only one linker DNA(s) is also unresolved^15^. It was also reported that the globular domain was located inside the DNA gyres and interacted with H2A^16^. Thus, the position of linker histones in the nucleosome particle has remained controversial.

The in vivo positioning of nucleosomes and linker histones is dynamic. The aforementioned binding models of linker histones to nucleosome core particle were mainly inferred from the structural studies using nuclear magnetic resonance (NMR) spectroscopy and cryo-electron microscopy (cryo-EM), which reveal the static contact between linker histones and nucleosomes and often fail to represent the bona fide in vivo context. However, knowledge of the precise placement of linker histones on nucleosomes is essential to understand the structural role in chromatin and regulatory role in cellular functions of linker histones.

However, it is a challenge for histone H1 study because H1 has many variants. For example, chicken have eight distinct variants, *Xenopus laevis* has five, both human and mouse have eleven^17^. This has largely limited progress in molecular, biochemical, and functional study on H1. In contrast, only a single linker histone H1 was detected in somatic cells through *Drosophila* embryonic development upon zygotic genome activation^18–20^. Therefore, *Drosophila* provides an ideal model to study H1.

We here generated genome-wide high-resolution maps of H1 and nucleosome positions in early *Drosophila* embryos at two stages. We applied epigenomics analyses to dissect the placement patterns of H1 on nucleosomes, how H1 binding affects nucleosome positioning, and the role of H1 in regulation of gene expression in vivo. Our study provides new insight into the binding patterns of linker histone H1 to nucleosome core particle, its roles in affecting chromatin structure and regulating cellular functions during early *Drosophila* embryonic develoment, from an epigenomics angle.

## Results

### H1 placement is positively correlated with nucleosome occupancy

**Fig. 1:**
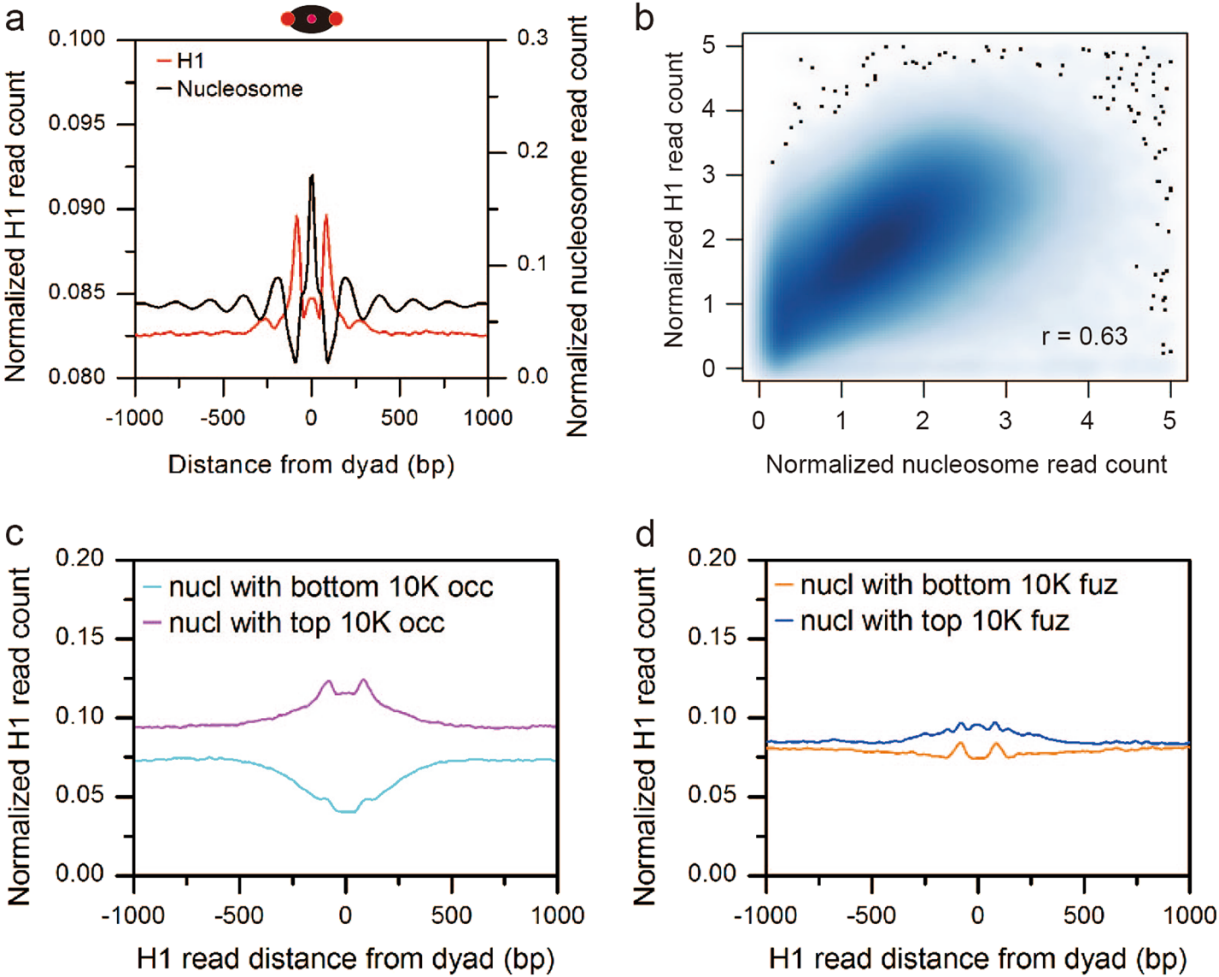
H1 profile around nucleosomes. **a** The red line shows the distribution of H1 around the nucleosome dyad. The black line showing the relationship between the position of adjacent nucleosomes. The right *y* axis shows the normalized nucleosome count with a dyad-to-dyad distance indicated on the *x* axis. The top schematic diagram shows the H1 (red) placement on nucleosomes (black oval). **b** H1 occupancy is positively correlated with nucleosome occupancy. **c** H1 locates at borders of nucleosomes with top 10,000 occupancy whereas H1 is depleted on nucleosomes with bottom 10,000 occupancy. All these nucleosomes have fuzziness of 30–35 to remove the impact of fuzziness. **d** H1 locates at borders and midpoint of nucleosomes with top 10,000 fuzziness whereas H1 only locates at borders of nucleosomes with bottom 10,000 fuzziness. All these nucleosomes have normalized read count of 0.8–1.2 to remove the impact of occupancy

In order to explore the pattern of H1 location on nucleosomes and the impact of its placement on nucleosome positioning, we generated genome-wide maps of H1 and nucleosome positions with mononucleosomal resolution in *Drosophila* embryos at 3–4 h after egg laying (AEL). The distribution of H1 around nucleosome dyad shows that H1 frequently locates on both borders of nucleosomes as well as much less frequently on the nucleosome middle point (Fig. 1a).

We next examined the impact of H1 occupancy on nucleosome occupancy. Occupancy measures the intensity of protein binding signals. Here, it is positively proportional to the number of sequencing reads defining a H1 or nucleosome location. The results show that globally H1 occupancy is positively correlated with nucleosome occupancy (Fig. 1b).

Fuzziness is another important feature of nucleosome positioning. It is defined as the standard deviation of all read coordinates that contribute to a nucleosome location and measures how delocalized a nucleosome position is^21^. Two nucleosomes with the same occupancy may have different fuzziness, vice versa. The result shows that nucleosome fuzziness ranges from 5 to 70. We grouped nucleosomes by fuzziness interval of 5 so that fuzziness difference is ignorable within the same group of nucleosomes. More than 85% of nucleosomes have fuzziness of 30–35 (24.8%), 35–40 (41.1%), 40–45 (19.2%) (Supplementary Figure 1a). We took the nucleosomes with top and bottom 10,000 occupancy from these three groups of nucleosomes, respectively. The H1 occupancy level is higher on nucleosomes with top 10,000 occupancy than those with bottom 10,000 occupancy in each group. Moreover, there is a prominent H1 on both borders of nucleosomes with top 10,000 occupancy whereas H1 is depleted on nucleosomes with bottom 10,000 occupancy. Notably, H1 emerges in the middle point of nucleosomes with top 10,000 occupancy as fuzziness increases (Fig. 1c and Supplementary Figure 1b, c). This confirms the positive correlation between H1 occupancy and nucleosome occupancy.

Similarly, we grouped nucleosomes by normalized read count interval of 0.4 so that occupancy difference is ignorable within the group. More than 54% of nucleosomes have occupancy of 0.8–1.2 (17.9%), 1.2–1.6 (19.1%), 1.6–2.0 (17.4%) (Supplementary Figure 1d). We took the nucleosomes with top and bottom 10,000 fuzziness from these three groups of nucleosomes, respectively. The H1 occupancy level is close between nucleosomes with top and bottom 10,000 fuzziness in each group. Moreover, there is a prominent H1 in the middle point of nucleosomes with top 10,000 fuzziness whereas H1 is absent in the middle point of nucleosomes with bottom 10,000 fuzziness (Fig. 1d and Supplementary Figure 1e, f). Further correlation analysis suggests that H1 occupancy is not correlated with nucleosome fuzziness (Supplementary Figure 1g).

We also examined the relationship between H1 fuzziness and nucleosome occupancy, and found no correlation between them (Supplementary Figure 1h). In contrast, H1 fuzziness is positively correlated with nucleosome fuzziness (Supplementary Figure 1i).

### H1 binding contributes to maintaining regular nucleosome spacing

**Fig. 2:**
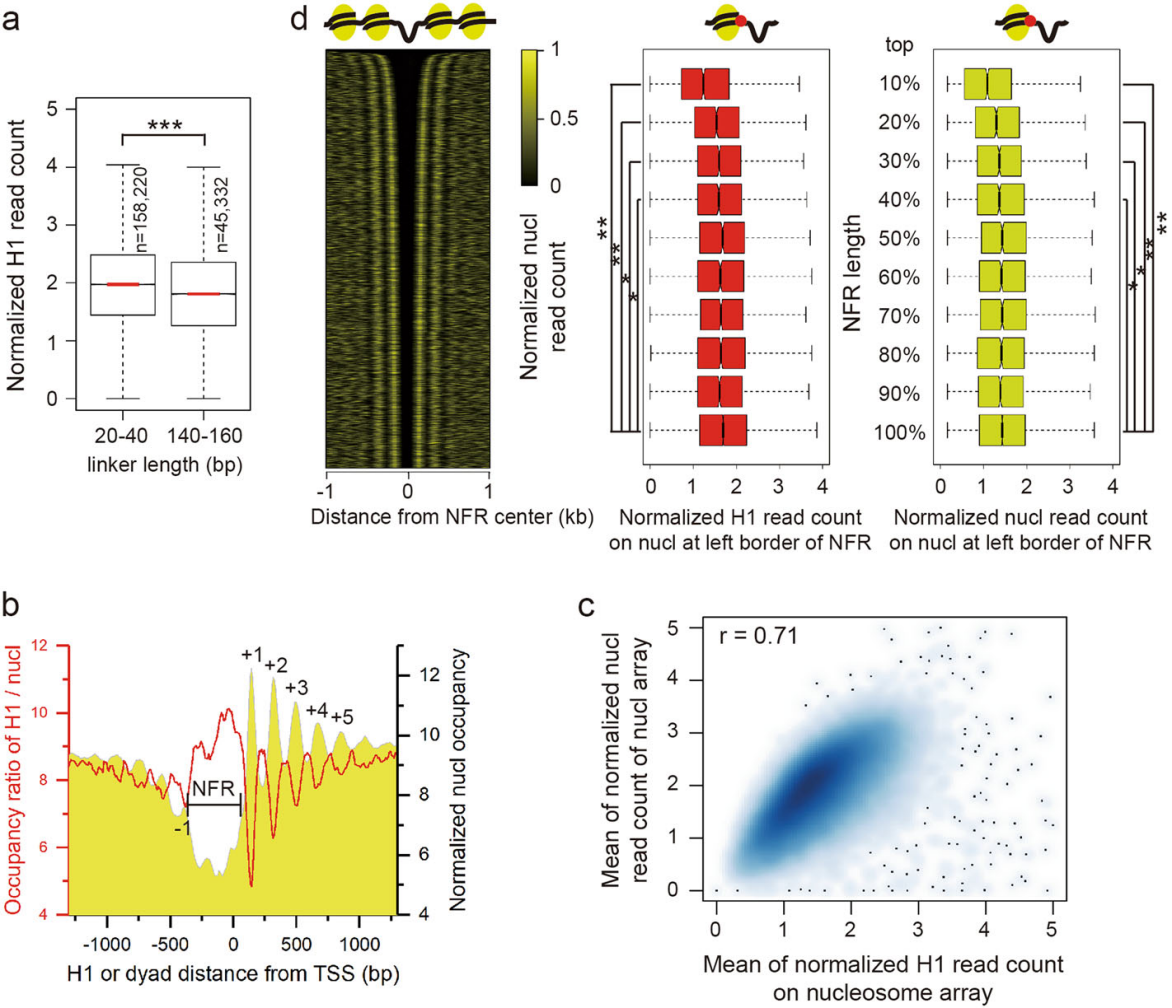
Correlation between H1 occupancy and nucleosome spacing. **a** H1 occupancy on short (20–40 bp) and long (140–160 bp) linkers corresponding to the bimodal peaks (see Supplementary Figure 2a) (***p-value < 0.001, two-sided permutation test). **b** Occupancy ratio of H1 to genic nucleosome in the regions closer to TSS is significantly lower than the farther downstream regions (*p* < 0.001, two-sided permutation test). The canonical -1, NFR, +1, +2, +3, etc. nucleosome organization around TSS is shown as a golden backdrop. **c** Scatter plot shows the positive correlation between H1 occupancy and average nucleosome occupancy of nucleosome arrays. **d** H1 occupancy is negatively correlated with NFR length. Left: heatmap shows the nucleosome organization around NFRs descendingly ordered by NFR length. Middle: H1 occupancy on the nucleosomes at the left border of NFRs grouped by NFR length. Right: Nucleosome occupancy on the nucleosomes at the left border of NFRs grouped by NFR length. H1 or nucleosome occupancy in the top four groups of NFRs is significantly lower than the group of NFR with the shortest length (*p-value < 0.05, **p-value < 0.01, two-sided permutation test). There are total 38,938 NFRs

Positioned nucleosomes in the genome are spaced at a fixed distance from each other by a short stretch of linker DNA between adjacent nucleosomes. The lengths of linker DNA vary from different organisms^22^. In consistence with our previous work^23^, the frequency of linker DNA length in *Drosophila* embryo peaks at 28 bp (Supplementary Figure 2a). Of note, there is a small peak at 150 bp. Therefore, we compared H1 occupancy between nucleosomes with linker DNA length of 20–40 bp and 140–160 bp and found H1 occupancy is significantly higher in nucleosomes with short linker DNA than in nucleosomes with long linker DNA (Fig. 2a).

To reveal the relationship between H1 occupancy and the nucleosome organization around the transcription start site (TSS), we examined the correlation between H1 occupancy level and its distance to TSS. To remove the impact of nucleosome occupancy, we calculated the occupancy ratio of H1 to nucleosome. The results show that the occupancy ratio is significantly lower in the regions closer to TSS than the farther downstream regions (Fig. 2b). Namely, the ratio is lower in +1 nucleosome than +2 nucleosome that is lower than +3 nucleosome, and so on. The ratio is lowest on the +1 nucleosome at the edge of the nucleosome free region (NFR). The high ratios in NFRs simply reflect the extremely lower level of nucleosomes indicating the low stability as well (Fig. 2b). This suggests that high H1 contributes to the maintenance of regular short nucleosome spacing in the canonical −1, NFR, +1, +2, +3, etc. nucleosome organization around TSS.

We next investigated the role of H1 in the nucleosome array organization since nucleosomes are arranged as a linear array as “beads on a string” in the genome. We identified 71,053 nucleosome arrays (defined as three or more continuous nucleosomes containing no NFRs). Intriguingly, H1 occupancy is positively correlated with the nucleosome occupancy of arrays (Fig. 2c and Supplementary Figure 2b). Taken together, the results imply that H1 placement contributes to the formation and stability of nucleosome arrays.

NFRs are often found at transcription start and end sites and play a critical role in regulation of gene expression. To investigate how H1 placement influences NFR formation, we then collected 100-bp or larger regions lacking nucleosomes as NFRs and examined H1 occupancy flanking NFRs. The results show that H1 occupancy on the nucleosomes at both borders of NFRs significantly decreases as the lengths of NFRs increase, same as nucleosome occupancy at both borders of NFRs (Fig. 2d and Supplementary Figure 2c). This suggests that depletion of H1 contributes to the formation of NFRs, and conversely high H1 occupancy could stop NFRs expanding.

### H1 binding is positively correlated with nucleosome stability

**Fig. 3:**
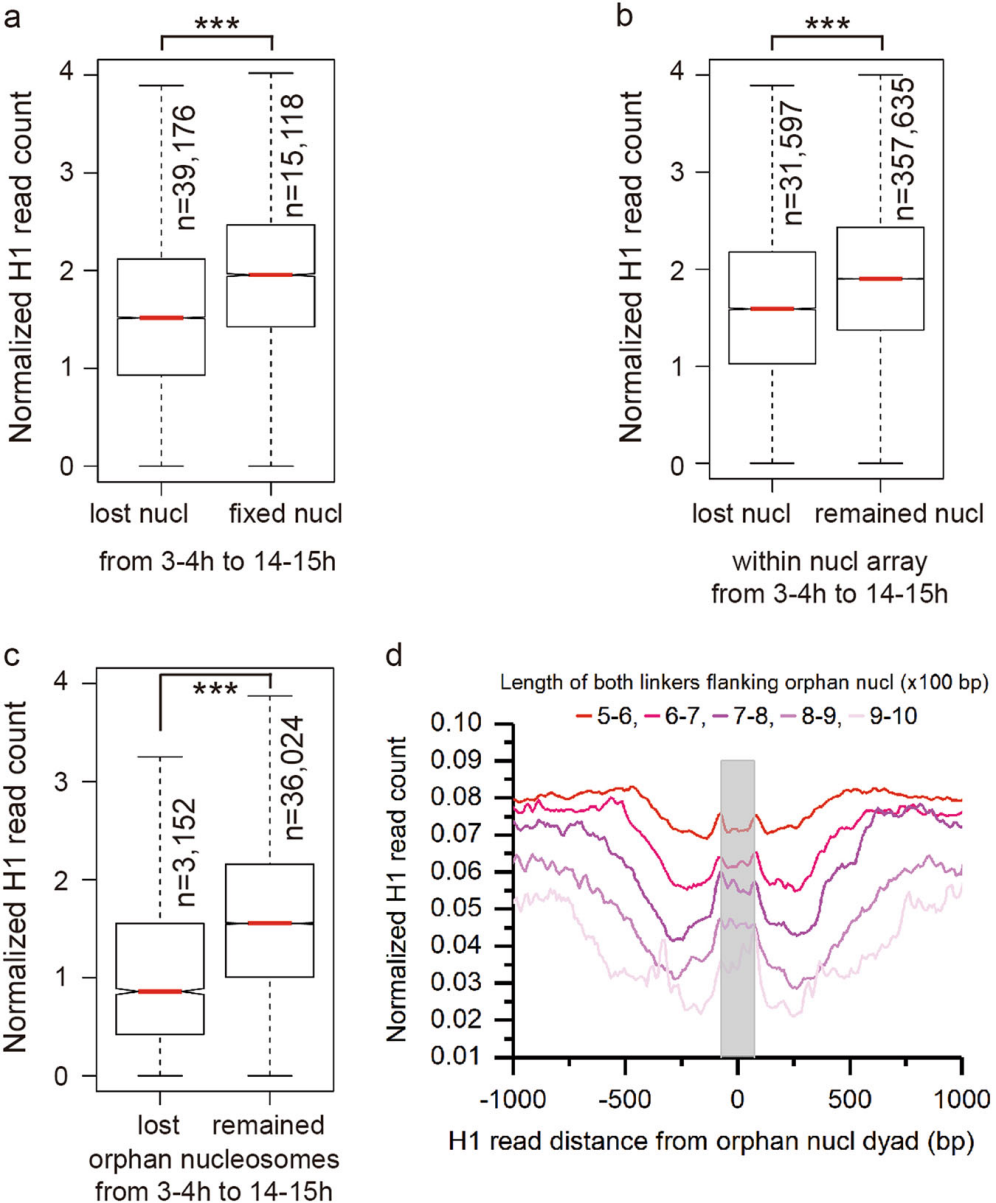
H1 binding is positively correlated with the stability of nucleosome positioning. Here shows H1 occupancy in 3–4 h embryos. **a** H1 occupancy on lost and fixed nucleosomes during the embryonic development from 3–4 h to 14–15 h (AEL) (***p-value < 0.001, two-sided permutation test). **b** H1 occupancy on lost nucleosomes belonging to a nucleosome array and the remaining component nucleosomes of nucleosome arrays during the embryonic development from 3–4 h to 14–15 h (AEL) (***p-value < 0.001, two-sided permutation test). **c** H1 occupancy on lost and remained orphan nucleosomes during the embryonic development from 3–4 h to 14–15 h (AEL) (***p-value < 0.001, two-sided permutation test). **d** Distribution of H1 around orphan nucleosome dyad categorized by the length of the both flanking linkers. The gray bar indicates the nucleosome location

Nucleosome remodeling plays a critical role in embryonic development. However, it has not been elucidated how H1 functions in dynamic nucleosome positioning during embryonic development. To address this, we compared the maps of nucleosome positions in *Drosophila* embryos at two stages (3–4 h and 14–15 h AEL), and identified fixed nucleosomes whose midpoint shifted ≤10 bp between the two stages, and lost nucleosomes whose midpoint shifted ≥127 bp from 3–4 h to 14–15 h AEL. H1 occupancy in 3–4 h AEL is significantly higher in the fixed nucleosomes than the lost ones (Fig. 3a). The result is the same for the component nucleosomes of nucleosome arrays. That is, H1 occupancy is significantly higher in the remained nucleosomes than the lost ones in nucleosome arrays (Fig. 3b).

We next focused on the orphan nucleosomes that were flanked by an NFR each side and were not in any nucleosome array by its definition (see above). As expected, H1 occupancy is significantly higher in the remained orphan nucleosomes than the lost ones from 3–4 h to 14–15 h AEL (Fig. 3c). Moreover, the overall H1 occupancy level of orphan nucleosomes is lower than non-orphan nucleosomes (not shown here). We further grouped orphan nucleosomes by the total length of their both linker DNA and profiled H1 occupancy in each group of orphan nucleosomes. The results show that H1 occupancy decreases as the total length of orphan nucleosomes’ both linker DNA increases. Moreover, the classical H1 placement on nucleosome borders vanishes in orphan nucleosomes with large linker DNA. Instead, H1 tends to randomly locate on orphan nucleosomes with large linker DNA (Fig. 3d). Together, this indicates that H1 binding contributes to the stability of nucleosome positioning.

### Skew placement of H1 on nucleosomes is predictive for nucleosome shift during early embryonic development

**Fig. 4:**
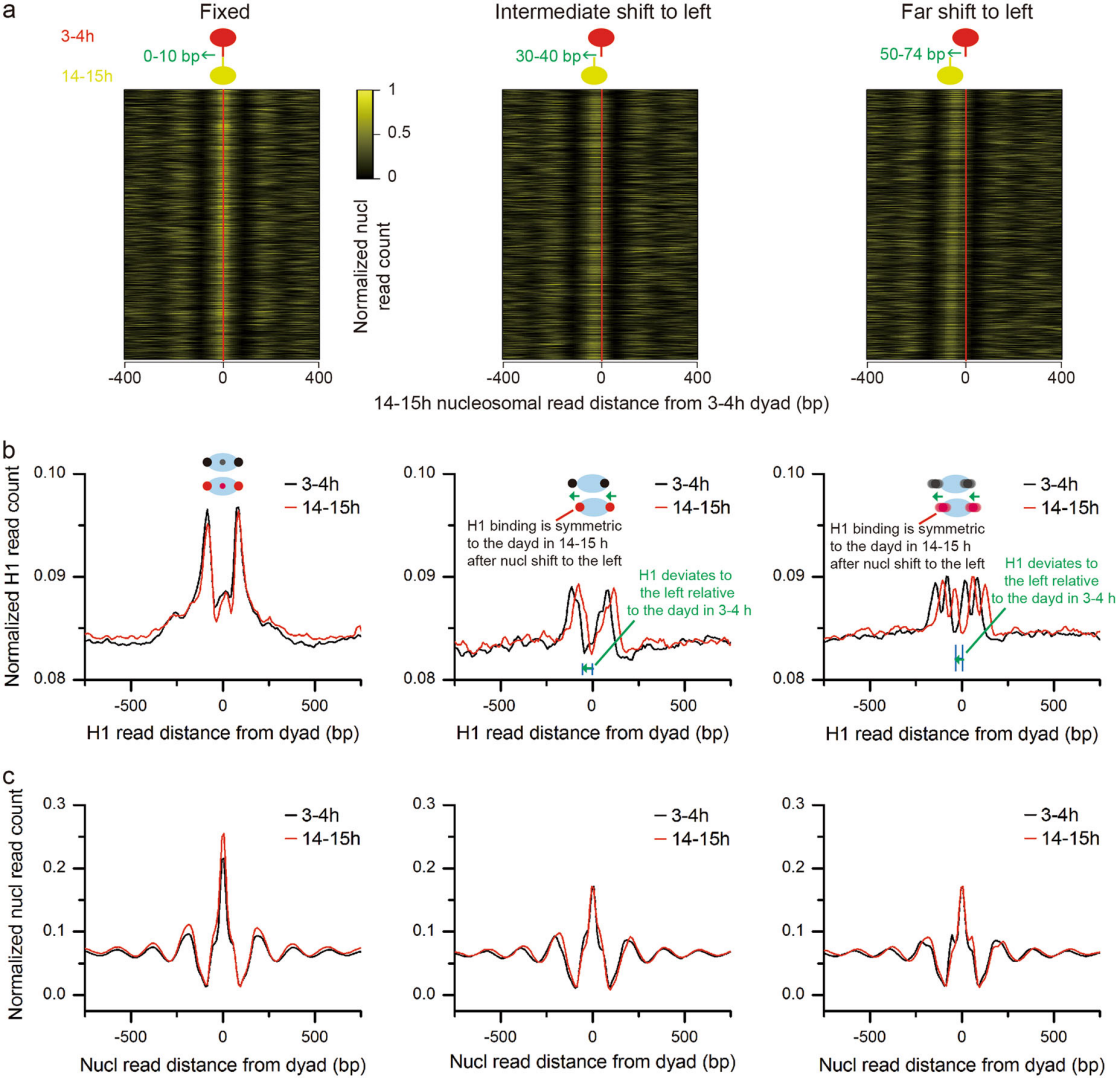
Skew H1 placement on nucleosomes predicts nucleosome shift direction. **a** Heatmaps shows three types of nucleosome dislocation from 3–4 to 14–15 h embryos (AEL): fixed (shift 0–10 bp), intermediate shift to left (30–40 bp), and far shift to left (50–74 bp). Gold indicates normalized nucleosome occupancy at 14–15 h (AEL) that is located relative to the dyad of the corresponding nucleosome at 3–4 h (AEL). **b** The distribution of H1 around the dyad of fixed (left), intermediate-shift-to-left (middle), and far-shift-to-left (right) nucleosomes. **c** Unchanged relationship between the position of adjacent nucleosomes at the two embryonic stages (left: fixed, middle: intermediate-shift-to-left, right: far-shift-to-left)

Shift is one of major nucleosome positioning dynamics. It remains unexplored how H1 impacts nucleosome shift. To address this issue, we identified all nucleosomes whose midpoint shifted up to 74 bp, half of a nucleosome, from 3–4 h to 14–15 h AEL. We collected three groups of nucleosomes: shift 0–10, 30–40, and 50–74 bp (termed as fixed, intermediate shift, and far shift) (Fig. 4a and Supplementary Figure 3a). H1 locates on the borders and on the dyad of the fixed nucleosomes. In contrast, H1 is absent at the dyad in the two groups of the shifted nucleosomes (Fig. 4b and Supplementary Figure 3b). Strikingly, nucleosomes with H1 placement deviating to the left relative to the dyad in 3–4 h AEL shift to the left when the embryos develop to 14–15 h AEL (Fig. 4b). Similarly, nucleosomes with H1 placement deviating to the right relative to the dyad in 3–4 h AEL shift to the right when the embryos develop to 14–15 h AEL. H1 placement becomes symmetrical to the dyad in 14–15 h AEL after nucleosome shift (Supplementary Figure 3b). Of note, H1 becomes two peaks on nucleosomes in the far shift group indicating higher fuzziness or possible alternative H1 positions (Fig. 4b and Supplementary Figure 3b). Further profiling analysis of nucleosome organization for these three groups of nucleosomes show an unchanged nucleosome array arrangement with regular spacing at both developmental stages (Fig. 4c and Supplementary Figure 3c). This rules out that the skew H1 placement is an artifact of skew nucleosome organization. Together, these findings suggest that skew H1 placement on nucleosome borders is predictive for nucleosome shift during *Drosophila* early embryonic development.

### Asymmetric H1 placement on nucleosomes in genic body associates with transcription direction and level

**Fig. 5:**
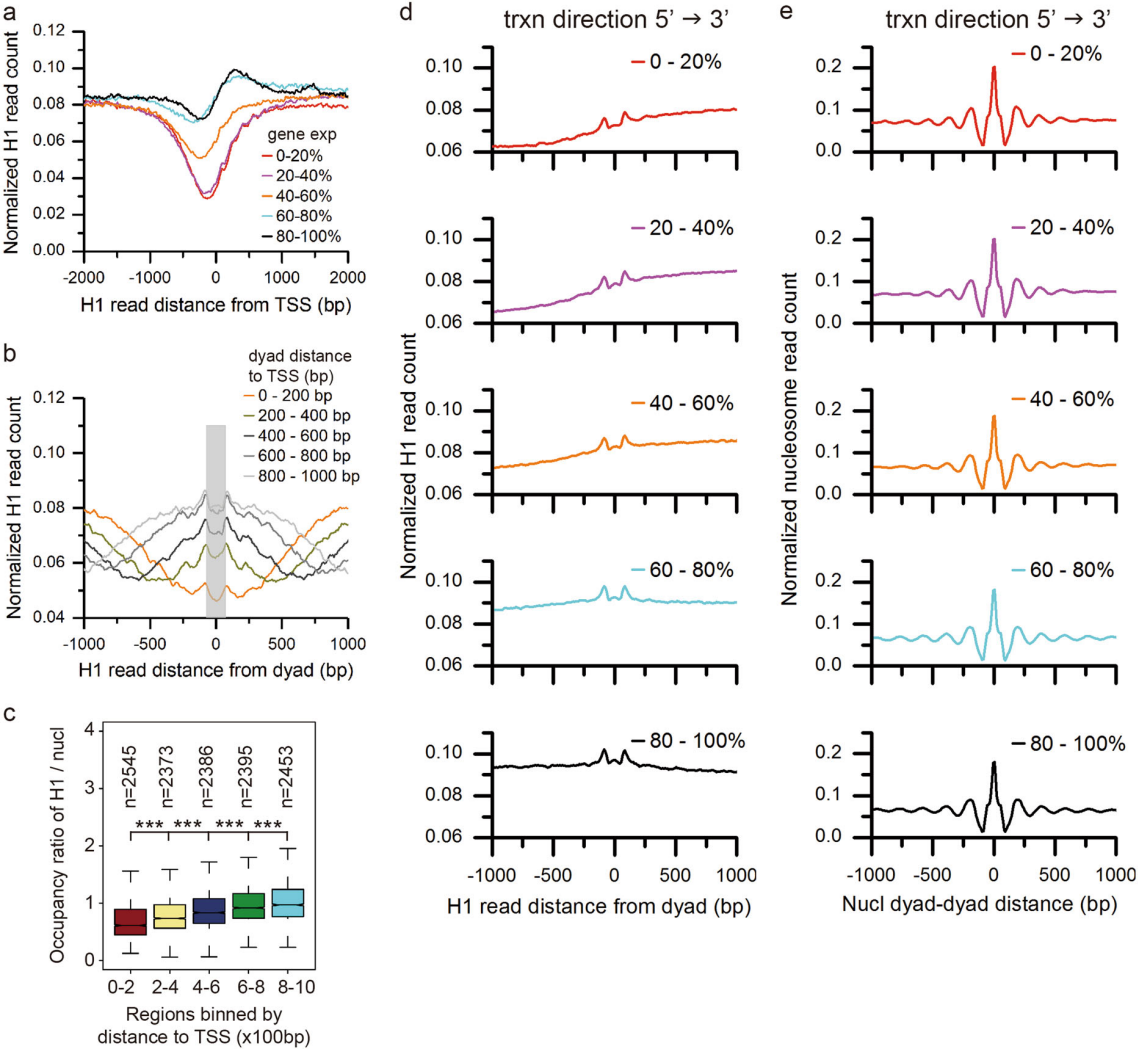
Gene expression-dependent asymmetrical H1 placement on nucleosomes in the genic body. **a** The distribution of H1 around TSS categorized by gene expression levels (top 20%, 20–40%, 40–60%, 60–80%, and 80–100%). **b** The distribution of H1 occupancy on genic nucleosomes grouped by the dyad distance to TSS with top 20% of gene expression levels. The gray bar indicates the nucleosome location. **c** Occupancy ratio of H1 to nucleosome in the regions closer to TSS with top 20% of gene expression levels is significantly lower than the regions farther to TSS (***p < 0.001, two-sided permutation test). **d** The distribution of H1 occupancy on genic nucleosomes between the 5′ and the 3′ end of genes whose length is <3 kb, or within 0–3 kb downstream of TSS of genes whose length is ≥3 kb. The nucleosomes are further classified by gene expression levels (top 20%, 20–40%, 40–60%, 60–80%, and 80–100%). Nucleosomes are orientated from 5′ to 3′ direction according the gene where the nucleosome locates. **e** Relationship between the position of adjacent nucleosomes corresponding to the groups in **d**. The *y* axis shows the normalized nucleosome count with a dyad-to-dyad distance indicated on the *x* axis

The nucleosome arrangement around TSS and in the gene body plays a critical role in regulation of gene transcription^22^. However, the organization of H1 around TSS and in the gene body and its relationship with gene activity remain enigmatic. To get insight into this question, we profiled H1 distribution around TSS grouped by transcription level. The result shows that there is a broad H1-depleted region closely upstream of TSS and predominant H1 occupancy in the gene body (Fig. 5a). The H1-depleted region is wide and deep in the highly active genes, and shrinks as the transcription level decreases. This implies that H1 occupancy around TSS and in the gene body is negatively correlated with gene transcription. Interestingly, a previous study also showed that the genes with H1.2 enrichment in the promoters tended to be repressed in human breast cancer cells^24^.

To understand how H1 occupancy impacts gene transcription, we profiled H1 occupancy on nucleosomes within the gene body. The H1 locates on the both borders of nucleosomes with its occupancy dependent on the nucleosome distance relative to TSS (Fig. 5b). The closer the nucleosome distance relative to TSS is, the lower H1 occupancy is. The correlation between H1 occupancy and the nucleosome distance to TSS weakens as gene transcription level decreases (Fig. 5b and Supplementary Figure 4a, b). We further normalized H1 occupancy by nucleosome occupancy to remove the interference of nucleosome occupancy. As expected, the occupancy ratio of H1 to nucleosome is also correlated with the distance to TSS. The closer to TSS the nucleosome is, the lower the occupancy ratio of H1 to nucleosome is. This correlation between H1 level and the nucleosome distance to TSS reduces as gene expression levels decrease (Fig. 5c and Supplementary Figure 4c, d).

All the above profiling of H1 placement didn’t take into account transcription orientation. Since H1 occupancy on genic nucleosomes was dependent on the distance to TSS we raised the question whether H1 occupancy on genic nucleosomes was also linked to transcription direction. To address this issue, we assigned the transcription direction of the gene to all the nucleosomes that located in this gene body. Then we calculated the composite distribution of H1 relative to the dyad of genic nucleosomes that were aligned from 5′ to 3′ end. Strikingly, H1 occupancy is asymmetric on the two borders of the nucleosomes within the gene body: lower at the 5′ border than the 3′ border (Fig. 5d). This asymmetrical pattern of H1 occupancy on genic nucleosomes is dependent on the transcription level. The asymmetry is prominent in the highly active genes, diminishes in the intermediately active genes, and vanishes in the lowest active genes. In contrast, nucleosomes are symmetrically organized in a form of array with regular spacing (Fig. 5e). This suggests that transcription level-dependent asymmetrical H1 placement on genic nucleosomes is not resulted from the unusual nucleosome organization. We further collected nucleosomes on genic bodies that were transcribed in one direction. These nucleosomes belong to the unidirectional units. We observed the skew placement of histone H1 on nucleosomes in the unidirectional transcription units (Supplementary Figure 4e). Similarly, we collected nucleosomes on overlapping genic bodies of two genes that were encoded in the plus and the minus strand, respectively. These nucleosomes belong to the bidirectional transcription units. The transcription direction of the gene with higher transcription level is used. There also exists the skew placement of histone H1 on nucleosomes in the bidirectional transcription units (Supplementary Figure 4f). Collectively, the asymmetrical placement of H1 on the genic nucleosomes is positively correlated with gene activity. Possibly, the lower H1 occupancy at the 5′ border of the genic nucleosomes provides a chromatin structure that is more accessible than the 3′ end and is consistent with transcription direction.

## Discussion

In this study, we determined the placement pattern of the linker histone H1 and its impact on nucleosome positioning and gene transcription during early *Drosophila* embryonic development. Our results show that nucleosomes with H1 placement deviating to the left relative to the dyad in 3–4 h AEL embryos shift to the left when embryos develop to 14–15 h AEL. Similarly, nucleosomes with H1 placement deviating to the right relative to the dyad shift to the right when embryos develop to 14–15 h AEL. Additionally, H1 occupancy on the genic nucleosomes is negatively correlated with the nucleosome distance to TSS. This negative correlation reduces as transcription levels decrease. Intriguingly, H1 occupancy is lower at the 5′ border of the genic nucleosomes than the 3′ border. This uneven pattern of H1 occupancy on the genic nucleosomes diminishes as transcription levels decrease. These findings greatly improve our understanding of the impact of histone H1 on nucleosome positioning dynamics and gene activity during early *Drosophila* embryonic development.

The location of the linker histone H1 in the chromatosome has remained controversial. Some studies suggested that the globular domain bound to the DNA located at the dyad and interacted with both linker DNAs^12,25–27^. Some studies showed that the globular domain bound to the DNA located at the dyad but interacted with only one linker DNAs^6,13,15^. Our study clearly demonstrates that H1 tends to position at the border of the nucleosome (Fig. 1a). This finding favors the model that the globular domain interacts with both linker DNAs. Moreover, H1 locates at the border of the nucleosome with a much higher frequency than at the dyad (Fig. 1a). This consists with the previous finding that human H1.4 linker histones bind to the nucleosome off the dyad^14^. However, this is also partially due to the fact that the C-terminal interacts with linker DNA^27–30^. Of note, both H1 and nucleosome position is dynamic. The aforementioned models of H1 binding were based on the structural studies using NMR and cryo-EM that represent the static structures in vitro. For example, the globular domain favors binding to the more rigid linker DNA on the most open chromatin conformations while the flexible linker DNA is selected for binding in a compact chromatin structure^31^. Although our epigenomics study using high-throughput sequencing failed to identify the structural domains and specific residues for the interactions between H1 linker histones and nucleosomal DNA, the results revealed the patterns of in vivo H1 binding within a chromatosome.

We show here that the left-skewed or right-skewed placement of H1 on nucleosomes is predictive for nucleosome shift during early *Drosophila* embryonic development (Fig. 4 and Supplementary Figure 3). Our results agree with the previous finding that H1 binding to the nucleosomal DNA has a strong inhibitory effect on nucleosome mobility^32^. When H1 binding deviates from the left border of the nucleosome, it creates space between H1 and nucleosome core particle. Consequently, nucleosomes shift to the left and H1 binding becomes symmetric to the dyad as *Drosophila* embryos develop next stage (Fig. 4). Similarly, nucleosomes shift to the right when H1 binding deviates from the right border of the nucleosome (Supplementary Figure 3). Thus the impact of H1 binding on nucleosome positioning alters chromatin structure. ATP-dependent chromatin remodeling enzymes are one of the important factors regulating nucleosome positioning. Knockdown of the chromatin remodeling complex Brahma extensively altered nucleosome positioning and led to the arrest of early *Drosophila* embryonic development^33^. Which chromatin remodeling complex, chromatin chaperone, and other factors drive skew binding of H1 in the first place and further interfere with the eventual shifting of the asymmetric H1-bound nucleosomes? More studies are needed to address these questions.

In addition to the structural role in chromatin, H1 also plays an important role in regulation of gene transcription. It has long been thought that H1 was a repressor of transcription. This view consists with the structural role in chromatin that the placement of H1 can inhibit nucleosome mobility and stabilize higher-order chromatin structure^32,34^. Consistently, our study shows that H1 occupancy is lower in highly-expressed genes than the lowly-expressed genes (Fig. 5b, c and Supplementary Figure 4a–d). Thus, the asymmetric distribution of H1 on the two borders of the nucleosome, low at the 5′ border and high the 3′ border, exists in the active genes not repressed genes (Fig. 5d). Of note, H1 appears to be not a repressor of global transcriptional activity in mammals. For example, with the involvement of DNA methylation, both increase and decrease in gene expression were observed in the H1 depleted mouse embryonic stem cells^7^. Deletion of H1 also down-regulated active euchromatic genes located in *Drosophila*^35^. Collectively, dynamics of H1 positioning regulates gene transcription through altering chromatin structure and with the involvement of DNA methylation in some cases.

## Materials and methods

### Embryo collection

Young (<5-day old) and healthy *Drosophila melanogaster* (w^1118^) were cultivated in two cages (45 × 34 × 34 cm, with 20,000–25,000 flies each) in an incubator at 25 °C and 55–60% humidity. Flies were fed on fresh grape juice plates with yeast paste for at least two days. The plates were replaced with new plates at least once a day. Prior to embryo collection, we replaced the plates with new plates. Exactly after one hour, these new plates that contained 0–1 h AEL embryos were removed from the incubator. Let the embryos continue to develop at 25 °C for 3 and 14 additional hours to get 3–4 h and 14–15 h AEL embryos, respectively. Then the embryos were collected from the plates and transferred into the mesh by a soft brush with PBST (137 mM NaCl, 4.3 mM Na_2_HPO_4_, 1.4 mM NaH_2_PO_4_, 0.01% Triton-X-100). Wash embryos with tap water to remove the yeast. Then embryos were immediately transferred into 100 ml beaker and dechorionated with bleach (5% sodium hypochlorite) for 3 min. Next, embryos were rinsed with tap water for 2 min and dried on paper. The embryos were ready for use.

### RNA-seq

Total RNA was extracted from embryos by TRIzol (Invitrogen). Genomic DNAs were removed with Turbo DNA-free kit (Ambion). The RNA sequencing libraries were constructed using standard Illumina libraries prep protocols. Sequencing was conducted on Illumina HiSeq2500 platform using 49-bp single-end protocol.

### MNase-seq and H1 ChIP-seq

The embryos were cross-linked immediately after collection with 1.8% formaldehyde in 3 ml of ChIP-fixed buffer (50 mM HEPES (pH 7.6), 100 mM NaCl, 0.1 mM EDTA, 0.5 mM EGTA) and 9 mL heptane on a shaker at 300 rpm for 15 min. Discard the supernatant after short centrifuge. Add 0.25 mM glycine with PBST to cease the cross-linking reaction. The cross-linked embryos were rinsed by PBST for three times, then were store at −80 °C for future use.

The cross-linked embryos were homogenized using Dounce All-Glass Tissue Grinders (885300-0007, Kimble) with A1 buffer (60 mM KCl, 15 mM NaCl, 4 mM MgCl_2_, 15 mM HEPES (pH 7.6), 0.5% Trition X-100, 0.1% NP-40, 0.5 mM DTT, 1 × EDTA-free protease inhibitor cocktail (PI, 04693132001, Roche)) on ice. The large tissue residuals were filtered by Miracloth (Calbiochem, cat.no. 475855) to get nuclei suspension. The mixture was centrifuged at 3500 rpm at 4 °C for 5 min. Then, the supernatant was discarded. The pellet was washed once with MNase digestion buffer (10 mM Tris-HCl (pH 7.5), 15 mM NaCl, 60 mM KCl, 1 mM CaCl_2_, 0.15 mM spermine, 0.5 mM spermidine, 1 × PI). The mixture was centrifuged at 3500 rpm at 4 °C for 5 min. Nuclei were suspended with 500 μl of 37 °C pre-heated MNase digestion buffer with 45 U MNase (Micrococcal nuclease, Cat.NO. LS004797, Worthington Biochemical Corporation) and incubated at 37 °C for 20 min. Reaction was terminated on ice by adding EDTA to a final concentration of 10 mM for 10 min. Then the mixture was centrifuged at 3500 rpm at 4 °C for 5 min. The pellet was washed with A2 buffer (140 mM NaCl, 15 mM HEPES (pH7.6), 1 mM EDTA, 0.5 mM EGTA, 1% Triton X-100, 0.1% sodium deoxycholate, 1 × PI). The pellet was resuspended in A2 buffer with 0.1% SDS. The pellet (i.e., nucleosomal DNA) was dissolved through sonication with three cycles of 20 s duration with at least 40 s pauses between cycles at the power setting of 6 (out of 20) on a Misonix sonicators XL-2000. The mixture was centrifuged at 13,000 rpm at 4 °C for 10 min. The pellet was discarded. Nucleosomal DNA was extracted by phenol–chloroform and dissolved in ddH_2_O.

In all, 20–25 μg of cross-linked genomic DNA was sonicated using XL-2000 Misonix sonicator with power output of 7 Watts. It is critical that the average length of the sheared chromatin is about 250 bp, with length ranging from 150–500 bp. The fragmented DNA was immunoprecipitated with anti-H1 antibody (Active Motif, 39575, Reactivity: *Drosophila*). The dose of anti-H1 antibody followed the specifications. ChIP reaction was done as described in the manual of ChIP kit (Cell Signaling 9003). In brief, the mixture containing chromatin, antibody, and ChIP buffer (16.7 mM pH 8.1 Tris-HCl, 167 mM NaCl, 1.2 mM EDTA, 1% Triton X-100, 0.01% SDS) was incubated overnight on a rotator at 4 ˚C. Then 30 μl of ChIP-Grade Protein G Magnetic Beads (Cell Signaling #9006) was added to IP reaction. The mixture was incubated for another 2 h with rotation. Then, beads were washed three times with low salt wash buffer (2 mM EDTA; 20 mM pH 8.1 Tris-HCl, 0.1% SDS, 1% Triton X-100, 150 mM NaCl) and once with high salt wash buffer (2 mM EDTA, 20 mM pH 8.1 Tris-HCl, 0.1% SDS, 1% Triton X-100, 500 mM NaCl). Five minutes for each wash. Removed wash buffer and added 150 μl of 1 × ChIP elution buffer (50 mM pH 8.1 Tris-HCl, 10 mM EDTA, 0.1% SDS) to suspend the beads.

The purified mononucleosomal DNA by MNase digestion and ChIP’ed H1 binding DNA were subjected to massively parallel DNA sequencing on Illumina HiSeq2500 platform using 49-bp single-end protocol.

### RNA-seq data analysis

Sequencing reads were aligned to the *Drosophila* transcripts (FlyBase r5.57) using TopHat (v 1.3.1) with default parameter setting. The uniquely mapped reads were assembled into transcripts guided by reference annotation with Cuffdiff (v 1.3.0)^36^ to calculate gene expression levels that were normalized as Fragment Per Kilobase per Million mapped fragments (FPKM).

### Nucleosome prediction

Sequencing reads were aligned to the *Drosophila* genome (dm3 assembly) using bowtie (v 0.12.7)^37^ allowing maximal two mismatches. The uniquely mapped reads were retained for nucleosome prediction. Each read was moved 73 bp interior to its end to represent nucleosome dyad. The nucleosome prediction tool GeneTrack^38^ was used to call nucleosomes with default parameters. Nucleosome read count were normalized by RPNM (reads per nucleosome per million mapped reads) as the nucleosome occupancy. Analysis was performed on only those 496,992 nucleosomes with RPNM value of 0.1 or higher, although virtually identical patterns (and conclusions) were achieved when all nucleosomes were included. Fuzziness were defined as the standard deviation of the coordinates of all reads defining the same nucleosome as described previously^33^. It measures how spread out a nucleosome position is. Each nucleosome was assigned to promoter, genic or intergenic regions depending on the location of the nucleosome midpoint.

### Definition of NFR, nucleosome array, and orphan nucleosome

Linkers with length >100 bp are defined as NFRs. Three or more continuous nucleosomes with no linkers with length >100 bp form a nucleosome array. Orphan nucleosomes are those that are flanked by an NDR on each side.

### Analysis of nucleosome positioning dynamics

The distance between the midpoints of the two closest nucleosomes from the two developmental stages measures nucleosome positioning dynamics. If the distance is greater than 127 bp, the nucleosome is dissembled or reassembled. If the distance is less than 10 bp, the nucleosome is fixed. The rest of nucleosomes are shifted.

Nucleosome organization in a region was plotted as heatmap as follows: nucleosome occupancy was z-scored to 0–1 and used as signal density. The dyad represents nucleosome position. Fuzziness represents nucleosome width.

### Profiling H1 placement on nucleosomes

H1 ChIP-seq reads were aligned to *Drosophila* genome as nucleosomal reads as above. H1 reads that overlap a nucleosome are assigned to this nucleosome. These reads were used to calculate occupancy and fuzziness of H1 as nucleosome’s as above.

The composite distribution of H1 around nucleosome dyads was calculated by aggregating H1 read count at each distance relative to the dyad as follows: each read represents an H1. We summed total H1 read counts at each site within ±1 kb of dyads of interest. The H1 occupancy equals to the read count normalized as RPNM. We further binned the H1 occupancy by a 10-bp interval of H1 read distance to the dyad, and smoothed it with 5-bin moving average and 1-bin step size.

The non-directed composite distribution of H1 around the TSS was calculated as H1 distribution around dyads as above.

The directed composite distribution of H1 around TSS differed in that all genic H1 was put in 5′-to-3′ direction before aggregating according to the transcription direction of the genes where H1 locates.

### Data accession numbers

The RNA-seq, MNase-seq and ChIP-seq data sets have been deposited in the Gene Expression Omnibus (GEO) under accession number GSE101330.

## Electronic supplementary material

SupplementalFile
