# Supplementary material for: Dynamic placement of the linker histone H1 associated with nucleosome arrangement and gene transcription in early Drosophila embryonic development
Source: Cell Death Dis. 2018 Jul 9;9(7):765. doi: 10.1038/s41419-018-0819-z (PMC6037678; doi:10.1038/s41419-018-0819-z)
Supplement: Supplementary file 1 — SupplementalFile [file 41419_2018_819_MOESM1_ESM.pdf]

Supplementary Figure Legends

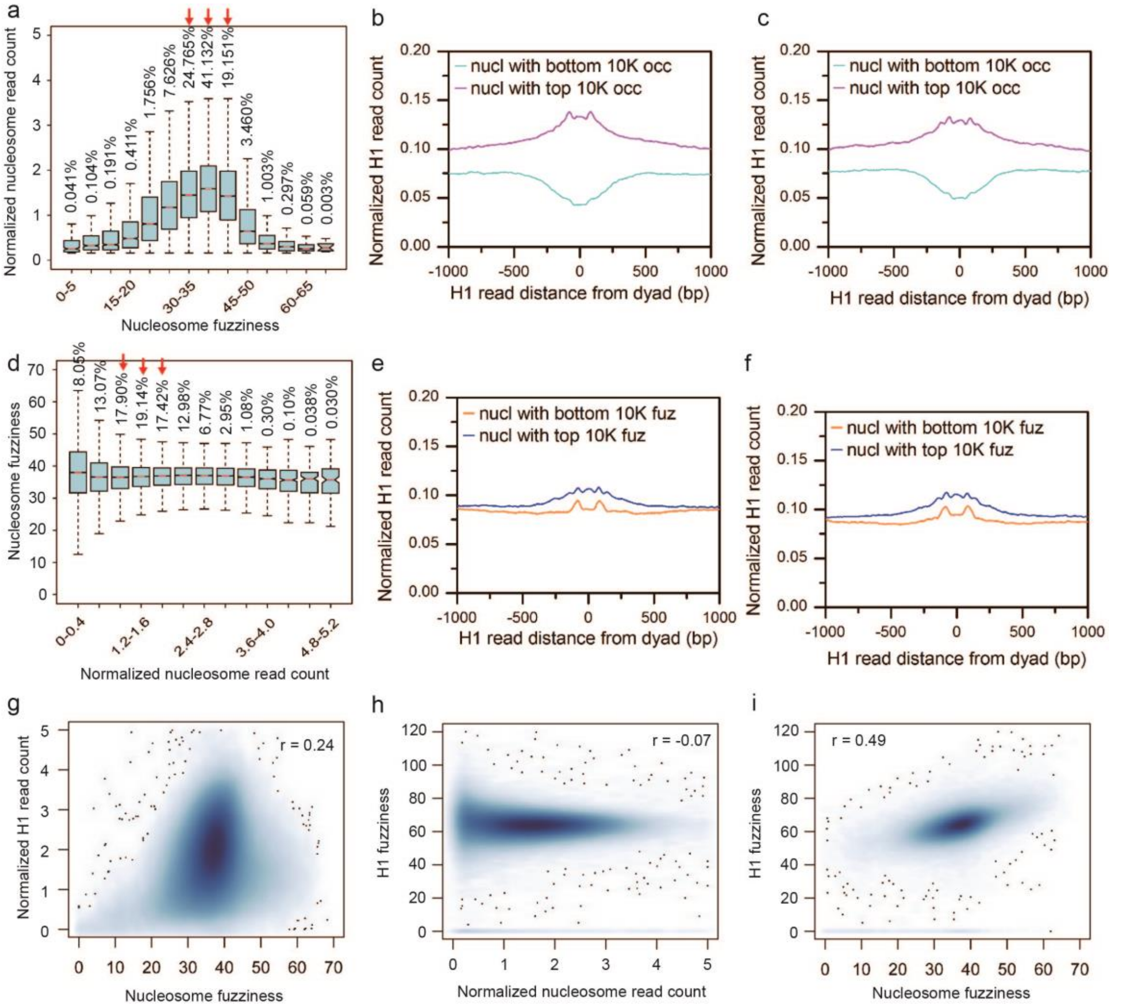

Supplementary Figure 1. Relationship between the positioning features of H1 and nucleosome.

(**a**) Occupancy frequency of nucleosomes categorized by fuzziness. The top numbers are the percentages of each category out of the total nucleosomes. Red arrows indicate the three largest groups of nucleosomes used in Figure 1c, Supplementary Figure 1b,c. (**b & c**) H1 locates at borders of nucleosomes with top ten thousand occupancy whereas H1 is depleted on nucleosomes with bottom ten thousand occupancy. All these nucleosomes have fuzziness of 35 - 40 (**b**) and 40 - 45 (**c**) to remove the impact of fuzziness. (**d**) Fuzziness frequency of nucleosomes categorized by occupancy. The top numbers are the percentages of each category out of the total nucleosomes. Red arrows indicate the three largest group of nucleosomes used in Figure 1d, Supplementary Figure 1e,f. (**e & f**) H1 locates at borders and midpoint of nucleosomes with top ten thousand fuzziness whereas H1 only locates at borders of nucleosomes with bottom ten thousand fuzziness. All these nucleosomes have normalized read count of 1.2 - 1.6 (**e**) and 1.6 - 2.0 (**f**) to remove the impact of occupancy. (**g**) H1 occupancy has no correlation with nucleosome fuzziness. (**h**) H1 fuzziness has no correlation with nucleosome occupancy. (i) H1 fuzziness is positively correlated with nucleosome fuzziness.

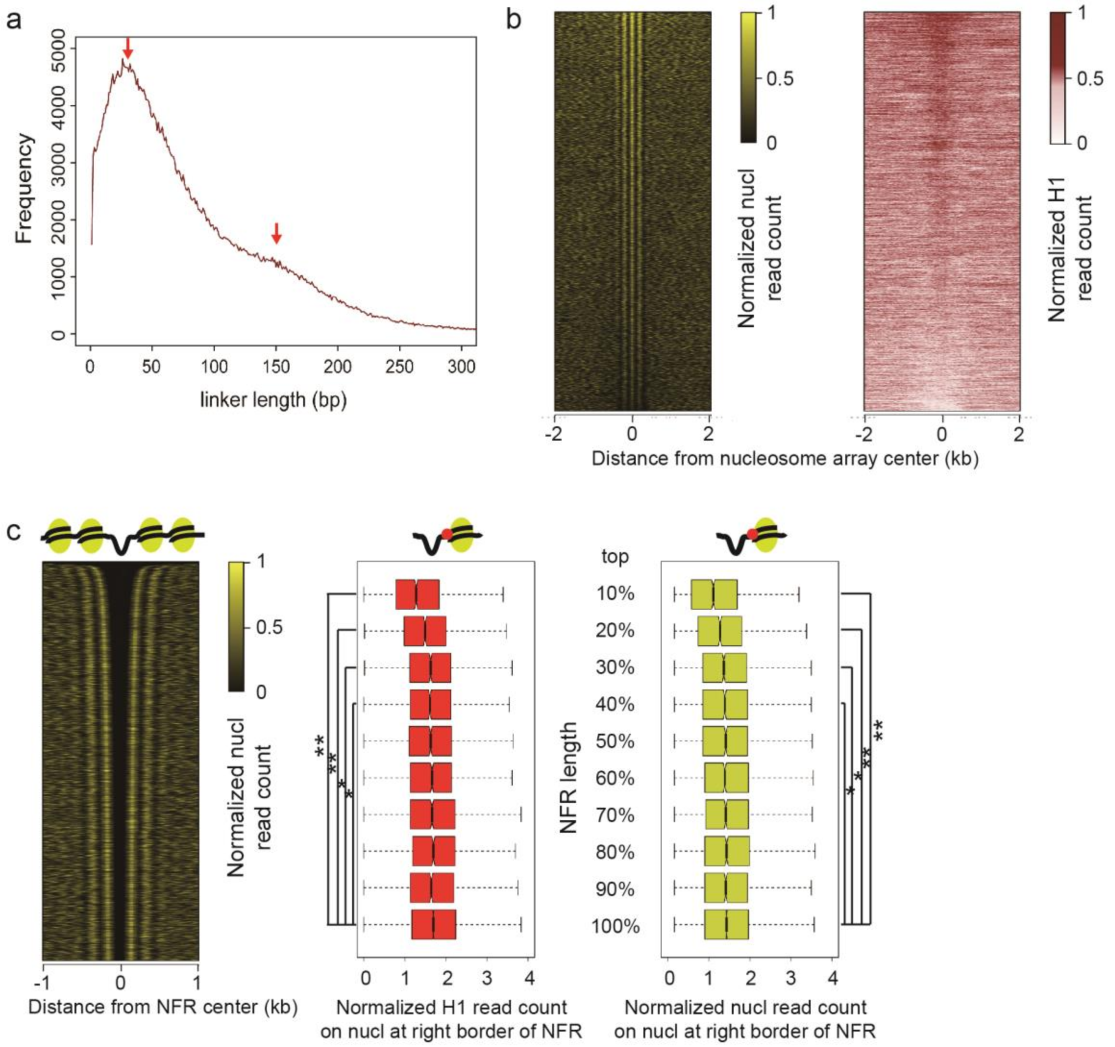

Supplementary Figure 2. Relationship between H1 occupancy and linker DNA length.

(**a**) Frequency of linker DNA length. Red arrows indicate the two peaks. (**b**) H1 occupancy is positively correlated with nucleosome occupancy of nucleosome arrays. Left: heatmap shows the nucleosome array organization descendingly ordered by the average nucleosome occupancy. Right: heatmap shows the corresponding H1 occupancy within the nucleosome arrays. (**c**) H1 occupancy is negatively correlated with NFR length. Left: heatmap shows the nucleosome organization around NFRs descendingly ordered by NFR length. Middle: H1 occupancy on the nucleosomes at the right border of NFRs grouped by NFR length. Right: Nucleosome occupancy on the nucleosomes at the right border of NFRs grouped by NFR length. Both H1 and nucleosome occupancy in the top four groups of NFRs is significantly lower than the group of NFR with the shortest length (*: p-value < 0.05, **: p-value < 0.01, two-sided permutation test). There are total 38,938 NFRs.

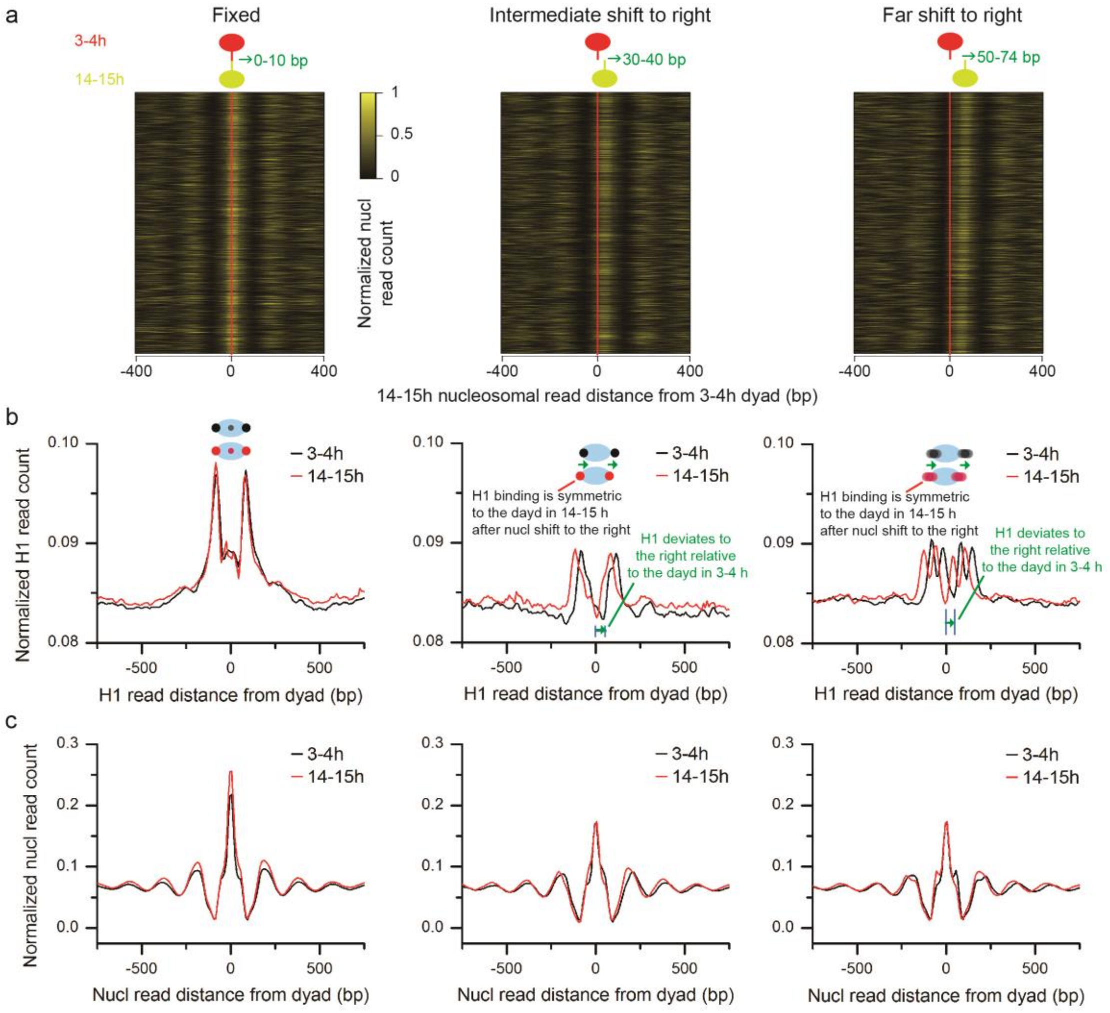

Supplementary Figure 3. Skewed H1 placement on nucleosomes predicts nucleosome shift direction.

(**a**) Heatmaps shows three types of nucleosome dislocation from 3-4 h to 14-15 h embryos (AEL): fixed (shift 0-10 bp), intermediate shift to right (30-40 bp), and far shift to right (50-74 bp). Gold indicates normalized nucleosome occupancy at 14-15 h (AEL) that is located relative to the dyad of the corresponding nucleosome at 3-4 h (AEL). **(b)** The distribution of H1 around the dyad of fixed (left), intermediate-shift-to-right (middle), and far-shift-to-right (right) nucleosomes. **(c)** Unchanged relationship between the position of adjacent nucleosomes at the two embryonic stages (left: fixed, middle: intermediate-shift-to-right, right: far-shift-to-right).

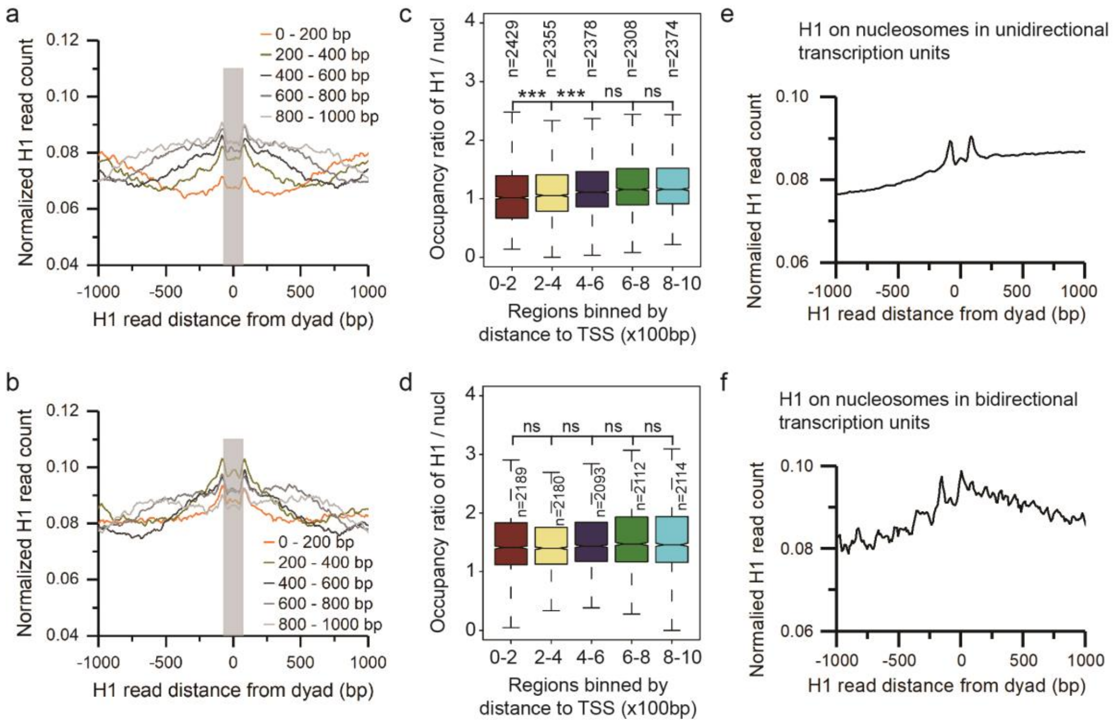

Supplementary Figure 4. H1 occupancy on genic nucleosomes is dependent on the dyad distance to TSS and the gene expression levels.

(**a & b**) The distribution of H1 occupancy on genic nucleosomes grouped by the dyad distance to TSS with top 40-60% (a) and bottom 20% (b) of gene expression levels. The gray bar indicates the nucleosome location. (**c & d**) Occupancy ratio of H1 to nucleosome in genic regions binned by the distance to TSS with top 40-60% (c) and bottom 20% (d) of gene expression levels. (***: $p < 0.001$, ns: not significant, two-sided permutation test) (**e**) The distribution of H1 occupancy on genic nucleosomes between the 5' and the 3' end of genes whose length is < 3kb, or within 0-3 kb downstream of TSS of genes whose length is ≥ 3 kb. These genic bodies are unidirectional transcription units. Nucleosomes are orientated from 5' to 3' direction according the gene where the nucleosome locates. (**f**) Similar as in (e) except that these genic bodies are bidirectional transcription units. The transcription direction of the gene with higher transcription level was used for the bidirectional transcription units.

Supplementary Table 1. Read count of RNA-seq, MNase-seq, and H1 ChIP-seq.

	Clean reads	Uniquely mapped reads	Mapping ratio (%)
3-4h RNA-seq	101082360	90012304	89.05
3-4h MNase-seq rep1	64744623	47072127	72.70
3-4h MNase-seq rep2	65312068	43414355	66.47
3-4h H1 ChIP-seq	81452587	55126501	67.68
14-15h MNase-seq rep1	80264469	58991919	73.50
14-15h MNase-seq rep2	70668559	48979771	69.31
14-15h H1 ChIP-seq	96848614	66662050	68.83

Note: 49-bp single-end sequencing by Illumina HiSeq 2000
